# Investigating machine learning and natural language processing techniques applied for detecting eating disorders: a systematic literature review

**DOI:** 10.3389/fpsyt.2024.1319522

**Published:** 2024-03-26

**Authors:** Ghofrane Merhbene, Alexandre Puttick, Mascha Kurpicz-Briki

**Affiliations:** Applied Machine Intelligence, Bern University of Applied Sciences, Biel/Bienne, Switzerland

**Keywords:** natural language processing, machine learning, eating disorders, mental health, artificial intelligence, anorexia, bulimia, binge eating

## Abstract

Recent developments in the fields of natural language processing (NLP) and machine learning (ML) have shown significant improvements in automatic text processing. At the same time, the expression of human language plays a central role in the detection of mental health problems. Whereas spoken language is implicitly assessed during interviews with patients, written language can also provide interesting insights to clinical professionals. Existing work in the field often investigates mental health problems such as depression or anxiety. However, there is also work investigating how the diagnostics of eating disorders can benefit from these novel technologies. In this paper, we present a systematic overview of the latest research in this field. Our investigation encompasses four key areas: (a) an analysis of the metadata from published papers, (b) an examination of the sizes and specific topics of the datasets employed, (c) a review of the application of machine learning techniques in detecting eating disorders from text, and finally (d) an evaluation of the models used, focusing on their performance, limitations, and the potential risks associated with current methodologies.

## 1 Introduction

Recent reports in broad media about the latest conversational chatbots, which can generate human-like texts in response to user questions have made natural language processing (NLP) famous to the broad public. Yet the possibilities of this field go far beyond text generation and chatbots. Classifying texts into two (or more) groups and automatically extracting indicators that suggest that a text snippet belongs to either of the groups is also a common task. In particular, when using machine learning, this allows the identification of patterns that might differ from what a human might detect that are nonetheless effective in separating the two groups.

Meanwhile, in clinical practice in mental health, inventories with scaling questions are often used for diagnosis. Such inventories have limitations, including for example defensiveness (the denial of symptoms) or social bias that can influence the results of the questionnaires (1). In these cases, an automated text analysis applied to specific open questions or interview transcripts can provide further source of information indicating the patient’s condition that is more resistant to manipulations such as those arising from defensiveness.

Defensiveness is common amongst those afflicted with eating disorders (EDs). Respondents to a survey investigating the denial and concealment of EDs (2) reported a variety of attempts to hide the respective ED. Furthermore, the authors of the study state that such methods were described as deliberate strategies. This makes it challenging to use clinical instruments where an inventory item contains obvious indications for which options to choose in order to obtain a specific result.

EDs generally occur in the form of unhealthy eating habits, disturbances in behaviors, thoughts, and attitudes towards food, causing in some cases extreme weight loss or gain. These disorders not only impact mental health but also have physical effects (3). EDs are classified in the category F50 of the ICD-10 and can refer to different disorders including anorexia, bulimia or overeating^1^. A study conducted by Mohler-Kuo et al. (4) in Switzerland discovered that the lifetime prevalence for any ED is 3.5%. Another survey investigating the lifetime prevalence of EDs in English and French studies from 2000 to 2018 found that the weighted means were 8.4% for women, and 2.2% for men (5).

The power of natural language processing (NLP) has already been applied to the field of mental health, especially in research. Feelings and written expression are closely correlated: An analysis of student essays has shown that students suffering from depression use more negatively valenced^2^ words and more frequently use the word “I” (6). Different approaches have been applied to explore how to use automated text analysis on tasks such as the detection of burnout (7), depression (8, 9), the particular case of post-partum depression (10, 11), anxiety (12), and suicide risk assessment (13), (14). Often, such methods are based on anonymized publicly available online data. Only little work makes use of clinical data. Furthermore, the English language has been the primary focus, even though these methods can be highly language-dependent, meaning that data and methods should be carefully reviewed when adapting to local languages. This is relevant, as it has been shown that adapting to the patient’s language is beneficial in mental health diagnostics and treatment (15). In our view, one aim of such technologies should be to explore ways to support clinical practitioners in their daily work, and provide them with additional sources of information to consider. Therefore, we often refer to such solutions as Augmented Intelligence^3^, rather than Artificial Intelligence, as they aim to empower humans rather than replacing them.

Despite existing work in the field of ML and NLP for depression, anxiety or suicide risk assessment, there has been a lack of a detailed systematic literature comparison on the automatic detection of EDs using NLP technologies for both clinical and non-clinical data. A recent survey (16) investigated the use of natural language processing applied to mental illness detection. The majority of the identified results (45%) had worked on depression, whereas only 2% were about eating disorders in general and 3% about anorexia. Whereas the broad scope of the survey provides a generous overview of the research landscape, it does not compare the case of eating disorders in detail.

In this paper, we have undertaken a systematic literature review to address this research gap, following the Preferred Reporting Items for Systematic Reviews and Meta-analyses (PRISMA) guidelines (17) to ensure a well-structured and transparent methodology.

We contribute to the field by (a) analyzing the metadata of published papers to understand the current trends and methodologies, (b) examining the sizes and targeted topics of the datasets used in these studies, (c) reviewing how machine learning techniques are applied to detect eating disorders from textual data, and (d) evaluating the performance, limitations, and potential risks of the models deployed in this domain.

Our research is guided by specific questions, structured around four distinct perspectives, which collectively form the core of our investigative approach.

- Demographical Questions (DemRQ): Focus on metadata aspects of the paper:
• DemRQ1: When was the paper published?
• DemRQ2: From which countries were the contributors of the papers included in this study?
- Input Questions (InputRQ): Focus on the format and topic of the input data:
• InputRQ1: Which languages were taken into consideration?
• InputRQ2: What was the size of the dataset used?
• InputRQ3: Which data sources were used for data collection in the case of both clinical and non-clinical data?
• InputRQ4: What types of eating disorders were addressed in these studies?
- Architectural Questions (ArchRQ): Focus on the experimental architecture:
• ArchRQ1: Which feature extraction technique was used?
• ArchRQ2: Which machine learning techniques in the field of NLP have been used for ED detection?
- Evaluation Questions (EvalRQ): Focus on the evaluation aspects of the trained model:
• EvalRQ1: How did the model perform?
• EvalRQ2: What are the limitations and risks of the existing methods, and how can they be improved?

The article is structured as follows: First, we describe our methodology such as the study design and the paper selection process. We then describe the results of the literature search and describe the findings of our review. Finally, we summarize our results and describe perspectives for future research in the field.

## 2 Methods

### 2.1 Study design

To answer our research questions, we conducted a structured literature review (SLR) following the Preferred Reporting Items for Systematic Reviews and Meta-analyses (PRISMA) guidelines (17). This includes standards for literature search strategies and setting criteria for the inclusion or exclusion of gathered works in the final review.

### 2.2 Literature search strategy

In accordance with PRISMA standards, we have set an 8-year time span for searching for documents (2014-2022) related to our research scope. We consider the year 2014 mainly because Bellows et al. (18) conducted a study on automatically detecting binge Eating disorder using clinical data, which we deem to be the initial research in the field. We then compiled a list of all databases to be searched. The list included the following databases:

- Google Scholar
- IEEE Xplore
- Pubmed

In addition, in order to efficiently conduct our database search we have compiled a list of keywords and conditions. These keywords are relevant to the research topic of EDs and their detection using NLP and machine learning techniques. Furthermore, the list included specific terms related to social media and online social networks in order to enable the identification of studies that explore the use of social media for the early detection of EDs, which is an ongoing research interest. The final query is presented below:

(eating disorder OR anorexia OR binge eating OR bulimia OR overeating) *AND* (natural language processing OR NLP OR text mining OR inventories OR machine learning OR artificial intelligence OR automatic detection OR early detection OR social media OR online social network OR clinical).

**Figure 1:**
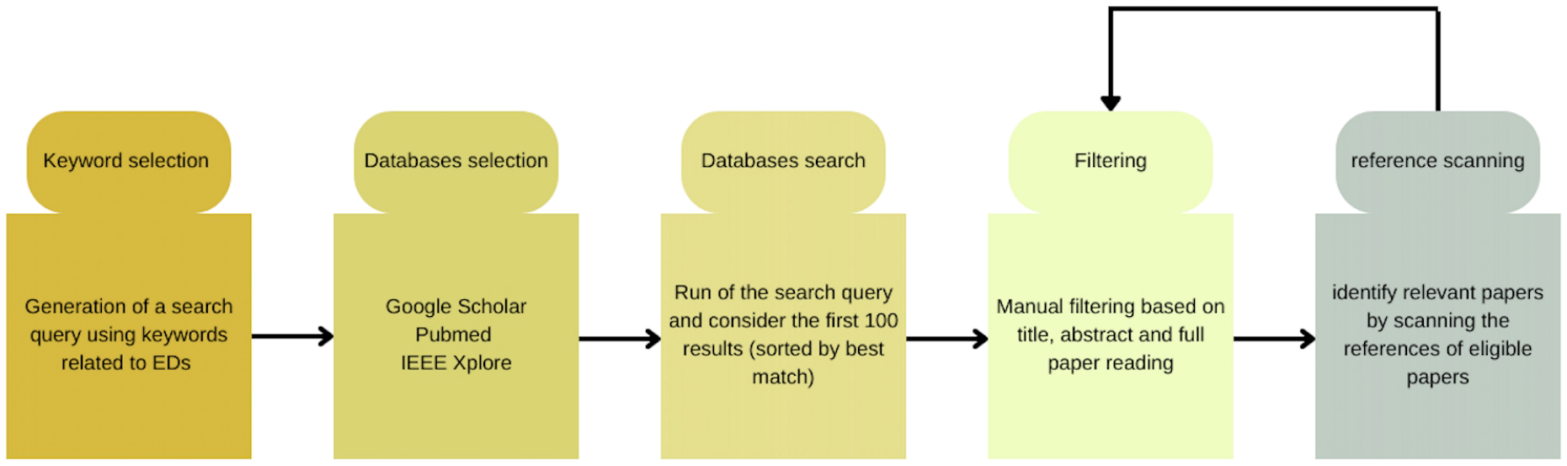
Methodology for document collection.

**Figure 2:**
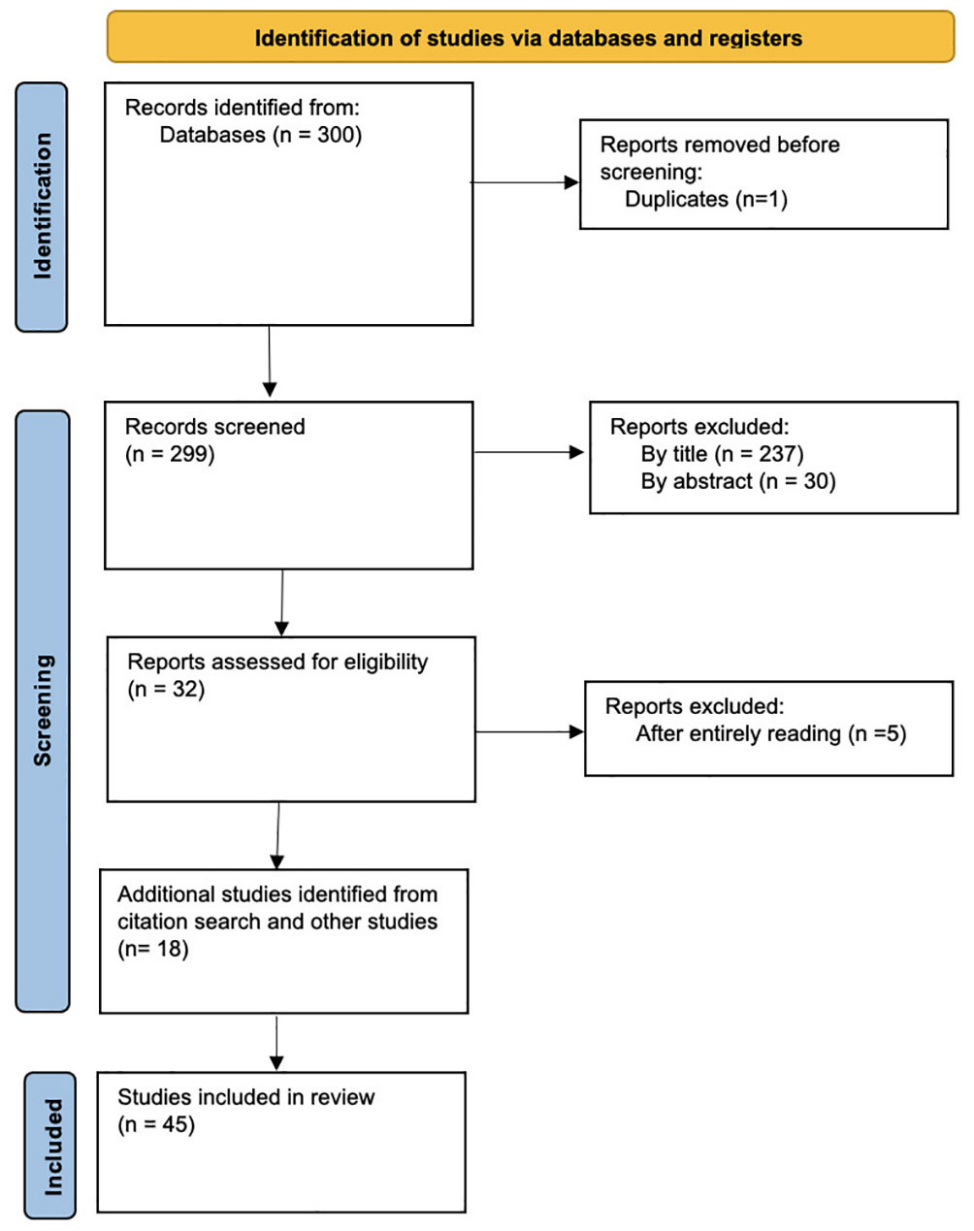
PRISMA Flow diagram. Based on: Page et al., (17).

Using the aforementioned search keywords and conditions, we retrieved research articles where NLP techniques have been used for the detection of EDs from clinical and non-clinical data. The detailed workflow is depicted in **Figure 1**, and the corresponding PRISMA flow diagram for this SLR is shown in **Figure 2**.

With the initially proposed search query, a large number of papers was identified. With manual analysis we explored options to define a more restrictive query, still making sure to capture the relevant papers, which turned out challenging. We therefore adapted our method to consider the first 100 elements returned by the search query on each database, sorted by relevance. This furthermore allowed to apply the same methodology for all three data sources, including especially Google Scholar, where the search functionalities are limited compared to databases like PubMed, and thus we had to make a selection on the number of items to be reviewed. Given the interidisciplinarity of our approach, we wanted to include Google Scholar to target a vast number of sources and ensure the most relevant work can be included.

A Python script was used to screen the articles for duplicates. As a result, 1 article was excluded from further consideration, leaving a total of 299 articles for further analysis (see **Figure 2**). To refine the results further, a manual title scan was performed to exclude articles that were not pertinent to the research topic. This resulted in the exclusion of 237 articles, leaving a total of 62 for further analysis. Additionally, a manual scan of the abstracts from the remaining 62 articles was performed to exclude any that were not relevant to the study. This process resulted in the exclusion of an additional 30 articles, leaving a total of 32 for inclusion in the final analysis. After thoroughly reading and evaluating 32 articles, 27 were selected as relevant for the researched topic (according to the criteria from **Table 1**). These chosen articles were deemed to possess high relevance and reliability for this SLR. Finally, we scanned the references section of the articles included in our survey and identified any relevant literature that may have been missed in the initial database search. This added n=18 articles to the studies that were finally included in the review (n=45). The process is illustrated in **Figure 2**.

**Table 1:** SLR study selection of literature using inclusion and exclusion criteria.

Criteria	Decision
When the predefined keywords exist in title, keywords or abstract section of the paper.	Inclusion
The paper should be written in the English language	Inclusion
When the paper targets other languages	Inclusion
Papers that are duplicated within the search documents	Exclusion
Papers that don’t make use of automated text analysis	Exclusion
Papers that deal with other types of data (than textual)	Exclusion
Papers that got published before 2014	Exclusion

### 2.3 Inclusion and exclusion criteria

**Table 1** outlines the predefined exclusion and inclusion criteria that were used to guide the selection of related studies for the review. These criteria were established in advance to help simplify the process of identifying and selecting relevant papers. In particular, papers that focused solely on the psychological aspects of EDs and did not consider the use of automated text analysis technologies were excluded from the review. By adhering to these criteria, we were able to more effectively and efficiently select the relevant papers.

## 3 Results

In this section, we provide a thorough review and analysis of the research studies included in this systematic literature review.

### 3.1 Terminology

- Bag of Words (BoW) is a fundamental technique used in NLP for text representation. It involves representing text data by counting the frequency of occurrence of each word in a document.
- Term Frequency-Inverse Document Frequency (TF-IDF) is a numerical statistic used to evaluate the importance of a word in a document within a collection or corpus. It combines two metrics: term frequency (TF), which measures the frequency of a word in a document, and inverse document frequency (IDF), which penalizes words that are common across the entire corpus.
- Bidirectional Encoder Representations from Transformers (BERT) (19) is a pretrained deep learning model introduced by Google in 2018. It belongs to the Transformer architecture and is designed to understand the context of words in a sentence by considering both left and right context simultaneously
- Word2Vec (20) is a technique for learning word embeddings. Word2Vec represents each word as a vector, with similar words having vectors that are closer together in the vector space.
- Global Vectors for Word Representation (GloVe) (21) is another technique for learning word embeddings. GloVe also generates vector representations of words based on their co-occurrence statistics in a corpus. However, GloVe considers the global context of the entire corpus to learn word embeddings, unlike Word2Vec, which focuses on local context.
- Embeddings from Language Models (ELMO) (22) is a deep contextualized word representation model. It generates word embeddings by considering the entire input sentence and capturing its contextual information.
- Doc2Vec (23) also known as Paragraph Vector, is an unsupervised learning algorithm to generate vector representations for pieces of texts like sentences and documents, it extends the Word2Vec methodology to larger blocks of text, capturing the context of words in a document.
- Bidirectional Long Short-Term Memory (Bi-LSTM) (24) is a type of Recurrent Neural Network (RNN) that processes data in both forward and backward directions. This architecture is particularly effective in understanding the context in sequence data like text or time series, as it captures information from both past (backward) and future (forward) states.
- Linguistic Inquiry and Word Count (LIWC) (25) is a text analysis program that counts words in psychologically meaningful categories.

### 3.2 Demographical research questions

**Figure 3:**
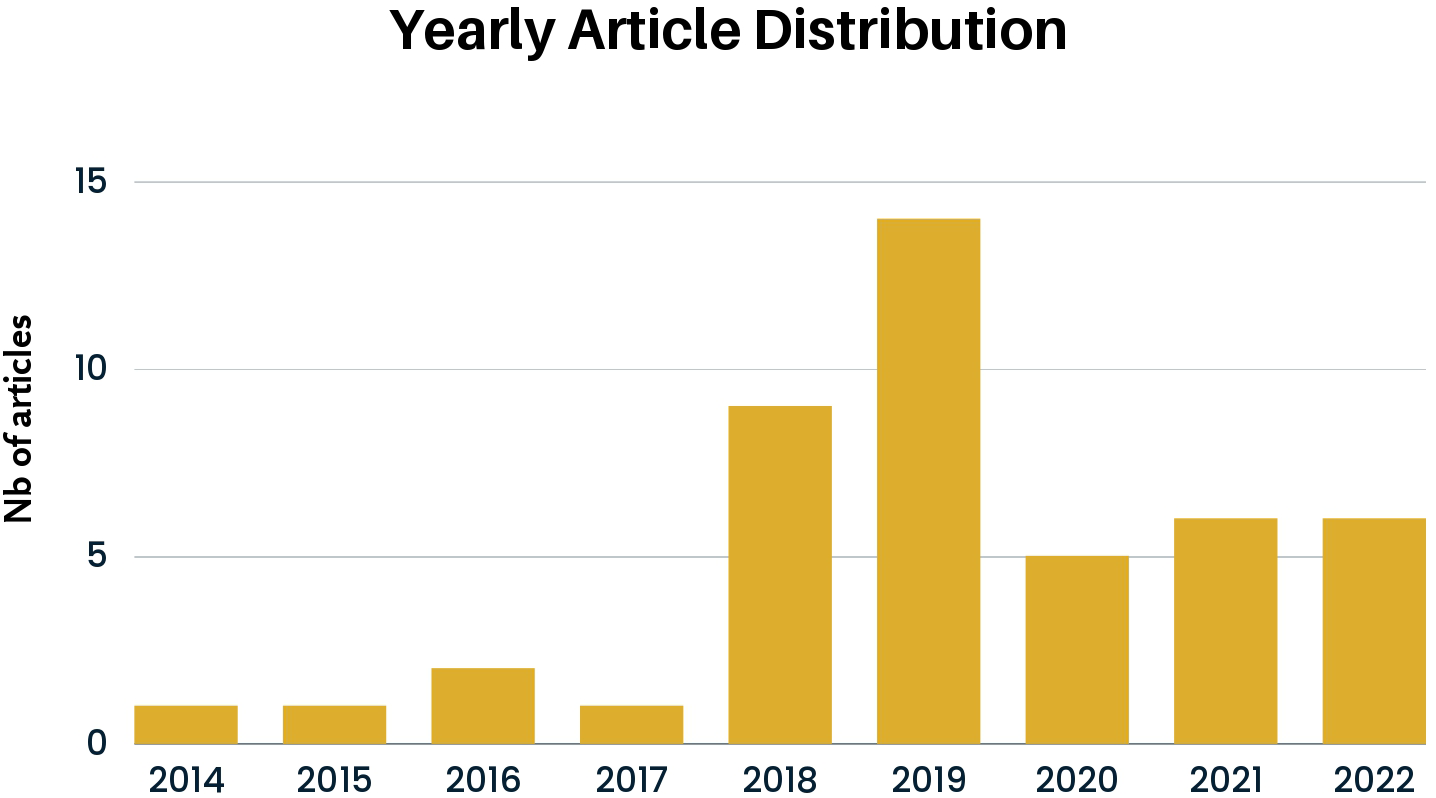
Yearly distribution of all research articles.

**Figure 3** shows the yearly distribution of the selected research work (DemRQ1). The data suggests a growing interest in this topic in recent years. This is in line with the findings of Zhang et al. (16) that found that there has been an upward trend over the last years in using NLP and machine learning methods to detect mental health problems. Notably, we highlight a prominent peak in 2018 and 2019, which coincides with the emergence of tasks related to EDs in eRisk competitions.

**Figure 4:**
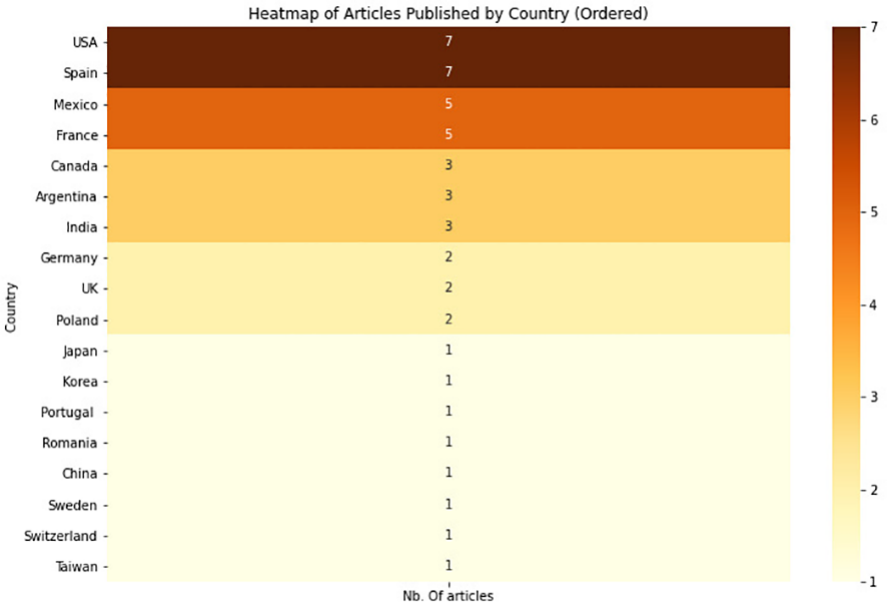
Geographic distribution of all institutions involved in the selected research articles.

We also observed the geographical distribution of the authors’ affiliations of the selected studies (DemRQ2). As visualized in the heat-map in **Figure 4**, 7 of the selected studies were from the USA and Spain, 5 from Mexico and France.

From the 45 selected studies, 24 were results from the eRisk lab^4^, hosted by the CLEF Conference since 2017. This academic research competition focuses on the development and evaluation of text-based risk prediction models for social media. Each year, the lab provides a shared task framework where teams of participants are tasked with developing NLP techniques to automatically identify and predict the risk of different mental illness behaviors from social media data, including Eating Disorders. Participants are provided with a training dataset and a test dataset, and the performance of their models is evaluated based on two categories: performance and latency. The eRisk lab provides a unique opportunity for researchers to collaborate and innovate in the field of NLP and mental health, aiming to improve the detection and prevention of mental health issues in online communities. The datasets used in the eRisk lab are primarily sourced from the social media platform Reddit.

Since 2017, the challenge has included two tasks pertaining to the early detection of Eating Disorders. In both 2018 and 2019, the task involved the early detection of signs of anorexia [see e.g., Losada et al. (26)]. In contrast, the 2022 iteration introduced a novel task centered on measuring the severity of eating disorders (27). This task diverged from the previous ones in that no labeled training data was supplied to participants, meaning that participants could not evaluate the quality of their models’ predictions until test time. The task objective was to assess a user’s level of eating disorder severity through analysis of their Reddit posting history. In order to achieve this, participants were required to predict users’ responses to a standard eating disorder questionnaire (EDE-Q)^5^ (28).

### 3.3 Input research questions

Our first input research question (InputRQ1) investigates the different languages that are considered in the studies included in this SLR. Research has shown that only a small number of the over 7000 languages used worldwide are represented in recent technologies from the field of natural language processing (29). We wanted to investigate whether this is also the case for the detection of eating disorders. Text analysis, naturally, depends on the specific language and can typically not be transferred from one language to another without specific adaptions.

**Table 2** gives indication about the language of data used, its size, its source, and the type of eating disorder that was investigated in the selected studies (excluding studies from eRisk). 18 of the 21 studies used English data, 2 used Polish and 1 Spanish data. The 24 papers from the eRisk lab challenges all relied on English data from the platform Reddit. Overall, only 3 out of 45 studies used a language other than English (7%). This confirms the need for further work in applying the latest technological developments to non-English texts.

**Table 2:** Datasets characteristics.

Paper	Language	Dataset Size	Data Source	Targeted ED
Choudhury (30)	English	10K-100K (55’334)	Social Media (Tumblr)	Anorexia
Yan et al. (31)	English	1K-10K (4’812 collected, 53 labelled by specialists)	Social Media (Reddit)	ED
BeníCheck that all equations and special characters are displayed correctly.tez-Andrades et al. (32)	English	1K-10K (1’085’957 collected, 2’000 manually labelled)	Social Media (Twitter)	ED
López Úbeda et al. (33)	Spanish	1K-10K (5’707)	Social Media (Twitter)	Anorexia
Zhou et al. (34)	English	1K-10K (123’977 collected, 2’219 manually labelled)	Social Media (Twitter)	ED
Aguilera et al. (35)	English	100k-1Mio (Dataset from 2018- 2019 editions of eRisk shared tasks)	Social Media (Reddit)	Anorexia
Spinczyk et al. (36)	Polish	*<*1K (96 written statements about the body image: 44 Anorexia females, 52 Healthy females)	Clinical Data	Anorexia
Aragon et al. (37)	English	*<*1K (Dataset from CLEF eRisk 2018 shared task)	Social Media (Reddit)	Anorexia
Bellows et al. (18)	English	1K-10K (1’000 Narrative Electronic Health Records)	Clinical Data	Binge Eating
Benítez-Andrades et al. (38)	English	1K-10K (1’085’957 collected, 2000 manually labelled)	Social Media (Twitter)	ED
Ramiandrisoa and Mothe (39)	English	100k-1Mio (Sequence of writings in chronological order of 472 users (eRisk 2019 data))	Social Media (Reddit)	Anorexia
Wang et al. (40)	English	*>*1Mio (119’825’361)	Social media (Twitter)	ED
He and Luo (41)	English	1K-10K (Tumblr 5’965 manually labeles) 100k-1000k (Twitter labeled based on hashtags)	Social Media (Tumblr and Twitter)	ED
Tébar and Gopalan (42)	English	100k-1Mio (253’341)	Social Media (Reddit)	ED
Dinu and Moldovan (43)	English	10k-100k (50’000)	Social Media [Reddit : Sample data from SMHD dataset from Cohan et al. (2018)]	MD^6^
Jiang et al. (44)	English	*>*1Mio (17.5m)	Social Media (Reddit)	MD
Zhang et al. (45)	English	1K-10K (8’554)	Social Media (Reddit)	MD
Hwang et al. (46)	English	1K-10K (3’714’057, 5’126 labelled)	Social Media (Reddit)	ED
Rojewska et al. (47)	Polish	*<*1K (51 written statements)	Clinical Data	Anorexia
Villegas et al. (48)	English	100k-1Mio (253’752)	Social Media (Reddit)	Anorexia
Chancellor et al. (49)	English	*>*1Mio (2’416’272)	Social Media (Instagram)	ED

**Figure 5:**
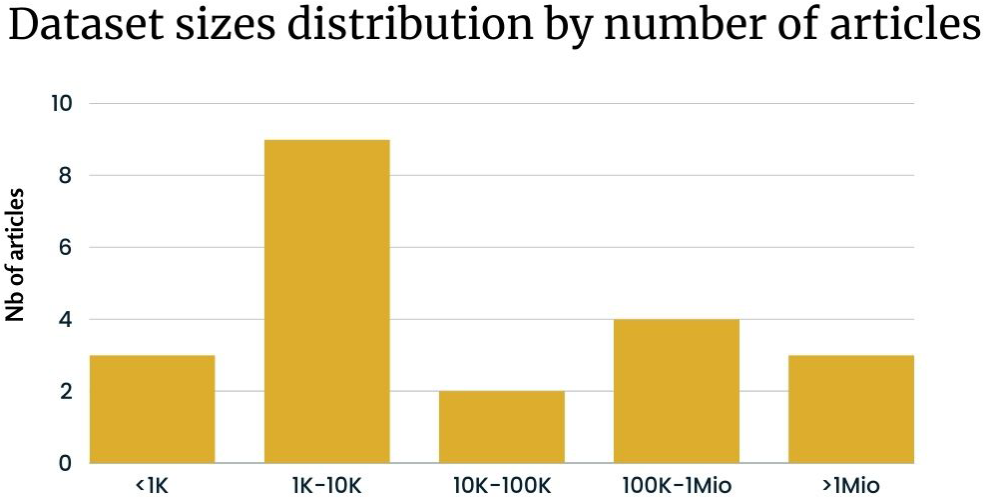
Dataset sizes distribution based on **Table 2** excluding articles from eRisk.

The dataset size is another crucial factor we took into account in our analysis ((InputRQ2). As depicted in **Figure 5**, the distribution of dataset sizes used in the studies reveals that datasets ranging from 1k to 10k instances are the most frequently used.

**Figure 6:**
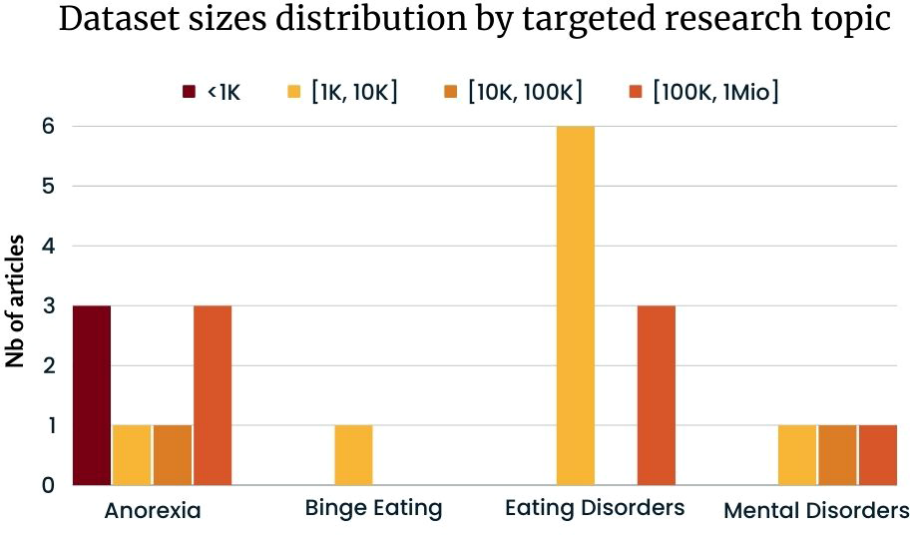
Dataset sizes distribution by targeted ED based on **Table 2** excluding articles from eRisk.

The distribution of dataset sizes across different research topics, as illustrated in **Figure 6**, offers insightful perspectives. Notably, Anorexia research displays the most significant variance in dataset sizes, spanning from less than 1K to over 1 million data points. In contrast, binge eating research predominantly employs datasets within a narrower range of 1K to 10K data points. For broader Eating Disorders, 6 studies leverage datasets between 10K and 100K, while 3 others operate with datasets in the 100K to 1 million range. Finally, research on Mental Disorders encompasses datasets varying from 1K to more than 1 million data points.

**Table 2** also gives an overview of the data sources (InputRQ3). From the 45 studies, the used datasets can be classified as follows in four groups:

- eRisk lab datasets: 24 studies
- Other online forums and social media: 17
- Medical data: 3
- SMHD dataset (50): 1

**Figure 7:**
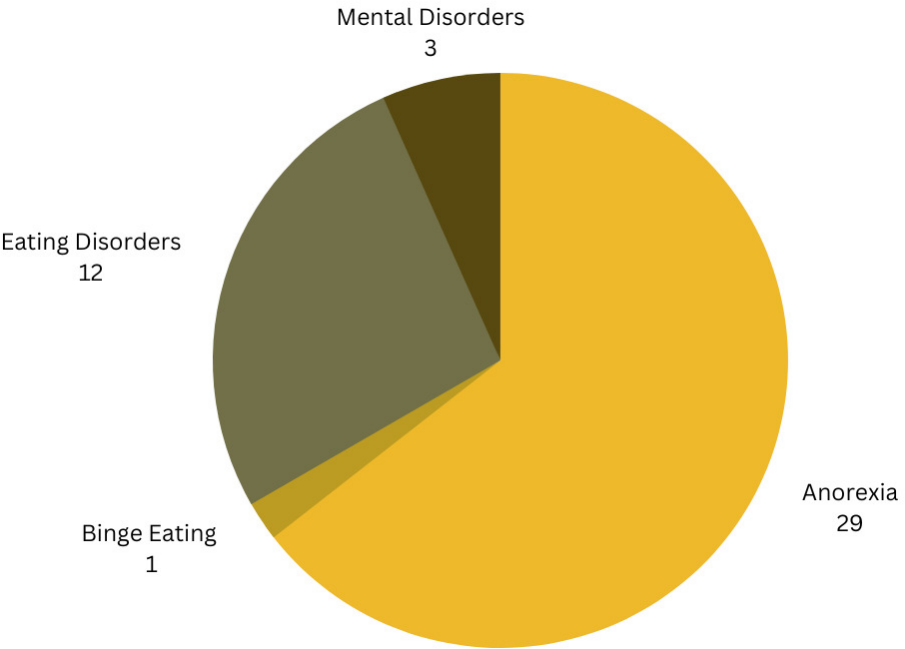
Research distribution of all research articles.

The distribution of the primary focus of these studies is illustrated in **Figure 7** (InputRQ4) The majority of the studies (n=29) we collected focused on anorexia, while 12 studies conducted a broader investigation of EDs in general rather than focusing on a specific type. Additionally, three studies had a more extensive scope, delving into various mental disorders, including but not limited to EDs, while one study focused on binge eating.

### 3.4 Architectural and evaluations research questions

#### 3.4.1 eRisk challenge

**Table 3** summarizes all the papers that we identified following our strategy, including the ones from eRisk. In 2018 and 2019, the eRisk papers focused on a text classification task aimed at developing an early detection system for eating disorders on social media using the history of users’ writings data. The aim was to train a text classifier that could effectively identify and flag potential cases of anorexia based on users’ social media content. For the eRisk challenge resulting in papers from 2022, the task was different. Participants were provided with the social media history of specific users and had to predict their answers to questions 1-12 and 19-28 from the Eating Disorder Examination Questionnaire (EDE-Q)^7^ (28).

**Table 3:** Overview of machine learning methods and performance metrics of the studies included in this systematic literature review.

Paper	Feature Extraction	Studied task	ML Techniques	Performance
Wang et al. (51)	**TF-IDF** for keyword selection and sentences encoded using the CNNbased sentence encoder	**Classification** (eRisk 2018)	Convolutional neural networks (CNN)	F1 score = 0.67
Paul et al. (52)	**BoW, UMLS** (Unified Medical Language System), and a combination of both	**Classification** (eRisk 2018)	SVM	F1 score = 0.67 with BoW
Trotzek et al., (53)	**Other** (Different techniques:**BoW/GloVe embeddings/fastText embeddings)**	**Classification** (eRisk 2018)	CNN	F1 score = 0.85
Ramiandrisoa et al. (54)	**Other** (Text vectorization using **doc2vec** (Two separate models were trained: 1- Distributed BOW with 100d output. 2Distributed Memory model wi9th 100-dimensional output))	**Classification** (eRisk 2018)	Logistic Regression	F1 score = 0.76
Ortega-Mendoza et al. (55)	**Other** (Discriminative personal purity (DPP), and a term weighting scheme called exponential reward of personal information (EXPEI))	**Classification** (eRisk 2018)	IG-EXPEI (a supervised classification model based on information gain and a term weighting scheme)	F1 score = 0.67
Ragheb et al. (56)	**Other** (Bi-LSTM Encoder)	**Classification** (eRisk 2018)	Bayesian inversion and Multi-layer Perceptron classifier	F1 score = 0.54
Liu et al. (57)	**TF-IDF**	**Classification** (eRisk 2018)	SVM, CNN+LSTM and a simple keyword model	F1 score = 0.36 for CNN+LSTM
Ramírez-Cifuentes and Freire (58)	**Other** (LIWC, anorexia vocabulary: 9 features and 1 weighted feature)	**Classification** (eRisk 2018)	Linear Regression	F1 = 0.73
Funez et al. (59)	**Other** (Sequential Incremental Classification (SIC))	**Classification** (eRisk 2018)	Sequential Incremental Classification (SIC)	F1 score= 0.60
Aragon et al. (60)	**Other** (Bag of Sub-emotions (BoSe))	**Classification** (eRisk 2019)	SVM	F1 score= 0.68
Burdisso et al. (61)	**Other** (Dictionary with a confidence value assigned to each work)	**Classification** (eRisk 2019)	SS3 (Burdisso et al., 2019a)	F1 score= 0.55
Ragheb et al. (62)	**Other** (Bi-LSTM Encoder)	**Classification** (eRisk 2019)	a Universal Language Model Fine-tuning for text classification with an additional attention layer	F1 score = 0.68
Fano et al. (63)	**Other** (GloVe)	**Classification** (eRisk 2019)	a Multilayer perceptron	F1 score = 0.68
Masood et al. (64)	**Other** (Term-frequency transformer + feature selection using chi-squared test to select the most significant 500 terms)	**Classification** (eRisk 2019)	SVM	F1 score = 0.61
Naderi et al. (65)	**TF-IDF**	**Classification** (eRisk 2019)	SVM	F1 score = 0.54
Mohammadi et al. (66)	**Other** (GloVe and ELMO (Both were used as submodels for an ensemble model for generating embeddings))	**Classification** (eRisk 2019)	SVM	F1 score = 0.71
Del Arco et al. (67)	**Other** (UMLS)	**Classification** (eRisk 2019)	SVM	F1 score = 0.30
Ranganathan et al. (68)	**Other** (Rapid automated keyword extraction (RAKE))	**Classification** (eRisk 2019)	CNN-LSTM (2-layer LSTM with normed-bahdanau attention)	F1 score = 0.34
Ferdowsi et al. (69)	**TF-IDF**	**Classification** (eRisk 2019)	CNN	F1 score = 0.17
Trifan and Oliveira (70)	**BoW** and **TF-IDF**	**Classification** (eRisk 2019)	SVM with SGD classifier	F1 score = 0.37
Ortega-Mendoza et al. (71)	**Other** (DPP-EXPEI (55))	**Classification** (eRisk 2019)	Linear SVM with L2 norm	F1 score= 0.58
Hosseini Saravani et al. (72)	**Other** (22 feature sets developed with expert knowledge and 300-dimensional word2vec and GloVe vectors of different sizes)	**Answer prediction** (eRisk 2022)	Cosine similarity	MAE = 3.15
Mármol-Romero et al. (73)	**Other** (RoBERTa contextualized word embeddings)	**Answer prediction** (eRisk 2022)	RoBERTa	MAE = 2.60
Srivastava et al. (74)	**Other** (Cosine Similarity)	**Answer prediction** (eRisk 2022)	BERT	MAE = 2.18
30	**Other** (Each data point is represented as a vector of four categories of measures: social, affective, linguistic style, and cognitive processes)	**Classification** (Binary: Detect anorexia content, differentiate between two online communities)	Binary SVM	F1 score= 0.818
Yan et al. (31)	**TF-IDF** (Bag of Bigram with TF-IDF reweighting) for trial 1-2, Word Embeddings (Word Mover’s Distance) for trial 3	**Classification** (Binary: Identify posts that require intervention as positive or negative)	Logistic Regression and Word Mover’s distance	Error Rate= 0.04
Benítez-Andrades et al. (32)	**BERT representations**	**Classification** (Binary: People that suffer(ed) from ED Vs. People that do/did not)	5 BERT based models	Accuracy= 0.875 for RoBERTa
López Úbeda et al. (33)	**TF-IDF**	**Classification** (people that suffer(ed) from anorexia vs. people that do/did not)	5 Different supervised learning models including: SVM, Multilayer Perceptron classifier, Naive Bayes, Decision Tree and Logistic Regression	F1 score= 0.91 for SVM
Zhou et al. (34)	**Word Embeddings** (Global Vectors for Word Representation pretrained 200-dimension Twitter word embeddings)	**Classification** (ED irrelevant, promotional information ED amd laypeople discussion ED)	Convolutional neural network (CNN), long short-term memory (LSTM), support vector machine, and Na¨ıve Bayes and CorEx for topic modelling	F1 score=0.90 for CNNLSTM and Coherence rate= 0.771 for topic modelling
Aguilera et al. (35)	**BoW** (1000 terms and TF weights) and average of the following word embeddings: 200- dimensions **GloVe** vectors trained on Twitter data, 300-dimensions **Word2Vec** vectors trained on the Google News dataset and 300- dimensions **FastText** trained on Wikipedia and on the UMBC and statmt.org news dataset	**Classification** (anorexia 1-class classification: The focus is only on instances that belong to the anorexia class).	One-class Classification kstrongest Strengths (OCCkSS) and Global Strength Classifier (gSC) both built based on the K-Strongest Strengths algorithm	F1 score= 0.671 with gSC
Spinczyk et al. (36)	**Word2Vec **100-dimensions vectors	**General sentiment analysis** from patient statements about their body images	Recurrent Neural Network (RNN) and Dictionary-based methods	F1 score= 0.70 for RNN and F1 score= 0.65 for Dictionary-based methods
Bellows et al. (18)	**Other** (Rule-based approach)	**Classification** (Identify binge eating Disorder Patients from EHR)	Not precise	Accuracy= 0.918
Benítez-Andrades et al. (38)	**Other** (Not precise)	**Classification** (Binary categories in 4 categorization tasks (People suffering from ED Vs. Rest, Tweets promoting ED Vs. Rest, Informative VS. Noninformative, Scientific tweets Vs. Rest)	Random forest, Recurrent neural networks, Bidirectional long short-term memory networks, Bidirectional encoder representations from transformer-based models	F1 score= 0.864 with RoBERTa
Ramiandrisoa and Mothe (39)	Method 1: **Other**: Feature-based text representation (Based on features extracted by the authors) Method 2: text vectorization using doc2vec.	**Classification** (Early detection of signs of anorexia)	Random Forest, Logistic Regression combined with word embedding text representation	F1 score= 0.71 for Random Forest and F1 score= 0.73 for Logistic regression
Wang et al. (40)	**Other** (Each user in the dataset was represented as a vector of 97 features obtained from the following measures: 6 social-status features, 11 behavioral features, and 80 psychometric features)	Snowball Sampling for Identifying Eating Disorder Communities on Twitter and a **Classification** (Binary: ED vs. NoED)	SVM	F1 score= 0.975
He and Luo (41)	**Other** (ADTree, a decision tree algorithm used to rank hashtags, the top 10 ranked hashtags were used as features)	**Classification** (Identify pro-ED posts on Tumblr and pro-ED users on Twitter)	CMAR (75).	Accuracy = 0.68 for identification of pro-ED posts on Tumblr and Accuracy= 0.92 for identification of pro-ED posts on Twitter
Tébar and Gopalan (42)	**Other** (Used topic modeling to get topics as features, frequency of ED-related words, and writing features (Nb. of words per post, time gap and Weekday/weekend posts and time of the day))	**Classification** (Early detection of signs of EDs)	Feature fusion Multimodal model	F1 score= 0.82 with BoSEunigrams
Aragon et al. (37)	**Other** (Used BoSE-based representations, and contrasted them against BoE and BoW schemes	**Classification** (Anorexia or depression vs. Control group)	SVM with a linear kernel	F1 score= 0.97
Dinu and Moldovan (43)	**Other** (used Naïve Bayes Classifier in order to find out the most informative features from each category in the dataset)	**Classification** of different mental illnesses including EDs	BERT, RoBERTa and XLNET	F1 score= 0.81 for BERT
Jiang et al. (44)	**Other** (LIWC (Used with logistic regression) and BERT representations (Used with an Attentionbased model)	**Classification** of different mental illnesses including EDs	BERT and REALM (76)	F1 score= 0.736 for BERT (post level classification)
Zhang et al. (45)	**BERT representations**	Build an annotated dataset for mental illnesses and **Classification** of these illnesses	BERT and MBERT (77).	F1 score= 0.51 for BERT
Hwang et al. (46)	**TF-IDF**	**Topic Modeling** (Analyze behavioral patterns of Emotional Eaters)	Stochastic gradient descent based ML model and LDA (Latent Dirichlet Allocation)	F1 = 0.91
Rojewska et al. (47)	**BoW** and Nencki Affective Word List	**Sentiment Analysis** and Emotion Detection	Recurrent Neural Network	–
Villegas et al. (48)	K-TVT, BoW, Word2Vec, GloVe and BERT representations	**Classification** (Early detection of signs of anorexia)	Naïve Bayes, Random Forest, Logistic Regression and SVM	F1 = 0.76 for BERT and Naïve Bayes
Chancellor et al. (49)	**Other** (Not precise)	**Topic Modeling** (Analyze the lexical variations and changes in pro-ED tags, and perform topic modeling on these tags)	Spectral Clustering algorithm	–

(ArchRQ1) The complexity of this task, along with the development in the field of NLP over the years 2019 to 2022, explains the choice of word2vec, GloVe (72) or transformer-based models (62, 66, 73) for vectorization/feature representation. For the remaining entries, very different approaches were used, ranging from anorexia specific vocabulary and LIWC (58) to more general approaches like Bag of Words (BoW) (52, 53) or TF-IDF (51, 57). (ArchRQ2) The choices of methods for prediction were also heterogeneous, ranging from cosine similarity (72) to linear models (52, 54, 58, 66, 71), to neural networks (51, 53, 56).

(EvalRQ1) For the 2018-2019 eRisk papers, we report F1 values corresponding to the binary classification task, whereas for the 2022 paper we report mean average error (MAE), corresponding to the average deviation between user’s predicted questionnaire responses and the ground truth responses.

#### 3.4.2 Non-eRisk studies

**Table 3** shows the feature representation, tasks studied, machine learning techniques, and performance metrics of all studies included in this SLR. In this section we focus on Non-eRisk studies. We grouped these studies into the following categories with regard to the feature extraction techniques they apply (ArchRQ1):

- Bag of Words (BoW)
- Word embeddings
- TF-IDF
- BERT representations
- and other feature representations

Furthermore, it is worth noting that the machine learning methods used in these studies span various categories (ArchRQ2), including:

- Classical machine learning (ML) methods such as Support Vector Machine (SVM), Naive Bayes, Logistic Regression, etc.
- Deep learning (DL) methods, e.g., recurrent neural networks.
- Combination of different methods from classical ML and DL.
- Large language models (LLMs), e.g., BERT.
- Other approaches.

Additionally, the tasks addressed in these studies can be broadly grouped into categories such as:

- Classification
- Topic modeling
- Sentiment analysis

In terms of feature extraction techniques employed across the 21 studies, a variety of methods were utilized. Among these, three studies (33, 46, 78) relied on TF-IDF. Four studies, including Zhang et al. (16) Benítez-Andrades et al. (38) Villegas et al. (48), and Jiang et al. (44), opted for BERT representations. Notably, Jiang et al. (44) combined BERT with LIWC.

Moreover, Bag of Words (BoW) and various types of Word Embeddings, including GloVe (35, 48), FastText (35), and Word2Vec (35, 36), were widely employed as feature extraction techniques in these studies.

It is pertinent to note that some studies, like Chancellor et al. (79) and Benítez-Andrades et al. (38), did not provide comprehensive details on this aspect in their papers. Conversely, other articles adopted a more personalized approach to construct their features. For instance, some represented each data point as a vector within certain categories (39, 40), while others used rule-based methods (18) or leveraged algorithms like decision trees (41) and topic modeling (42) to determine feature selection.

Our results show that from the 21 studies, 8 make use of classical machine learning methods, 1 uses deep learning, 5 use a combination of classical ML and DL, 4 use large-language models and 3 use other approaches.

When using classical machine learning, some studies compare different methods. For example, López Úbeda et al. (33) apply 5 different supervised machine learning models: SVM, multilayer peceptron classifier, naive bayes, decision tree and logistic regression, and Villegas et al. (48) compare naive bayes, random forest, logistic regression and SVM. Along with the classical machine learning methods, the studies apply different feature representations ranging from Bag of Words (BoW) to TF-IDF (33, 78), up to contextualized embeddings such as BERT (48).

Other studies compared both classical machine learning as well as deep learning methods. For example, in the case of Tébar and Gopalan (42), a so-called feature fusion model that includes both deep learning (a convolutional neural network (CNN) and a BiGRU model), as well as a classical machine learning model (logistic regression classifier with handcrafted features) is used.

For the studies using transformer-based large language models, different models including the BERT (19) model and its variations have been used. For example, Benítez-Andrades et al. (32) applied five variations of the BERT model. The paper from Dinu and Moldovan (43) uses BERT, RoBERTa and XLNET, whereas Jiang et al. (44) use BERT and REALM. The work from Zhang et al. (45) focusing on different mental illnesses used the BERT model, as well as the MBERT variation.

(EvalRQ1) The performance of each study is also reported in **Table 3**.

(EvalRQ2) Finally, we investigated the limitations of the proposed studies (RQ4) in order to provide a structured outlook for future work in the field.

In many cases, there were limitations in terms of the datasets. For example, Yan et al. (78) cites the limited availability of labeled data. They used a dataset of 50 posts, which they expect to be labeled correctly. Also Zhou et al. (34) mention that their study is limited by the number of collected tweets, which may result in some irrelevant topics arising from noise for their topic modeling task.

In many studies, social media data is used. The nature of such data is seen as a potential limitation for the resulting methods (37). Other studies indicated as a limitation that only one social media platform was used to gather their data (38, 42). For example, a study from (35) points out that their work did not take into account the potential biases in the data that may exist, such as underrepresented population or lack of diverse perspectives. In addition, one of the notable constraints arises from the fundamental disparity between social media data and traditional clinical text data, often used in healthcare and medical research. Clinical records encompass detailed information on patients’ medical histories, diagnoses, treatments, and outcomes, rendering them fundamentally distinct from the informal, user-generated content prevalent on social media platforms. Several studies point out that the involvement of clinical professionals would be beneficial. For example, Choudhury (30) states that their method could be more successful with the involvement of clinicians.

Different studies rely on anonymous data, which makes it difficult to ensure a good distribution within the training data over different populations and underrepresented groups. For example, Ragheb et al. (62) sees potential to optimize the model for different use cases and populations. Manual labeling by humans is also considered a source of bias since limited information about the users writing them is available to the annotators. This limited information may not encompass the full context of the users’ lives, beliefs, or backgrounds. Annotators may make subjective judgments based solely on the content of the post, which can be influenced by their own biases and interpretations. Thus, limited context can lead to misinterpretations or mislabeling, potentially distorting the research results (38).

In the limitations, it is also discussed how texts written by laypeople and ED promotional^8^ and educational materials can be hard to classify (34). This can be partly explained by the short length of texts, for example in the case of tweets, and the semantic similarity of the two types of texts.

Whereas many studies achieved good performance in terms of accuracy or f1-scores, they see a potential limitation in this matter. For example, Wang et al. (40) discusses that the validation was done only with a small sample of the data, and thus further validation is required with larger samples. In another study, the authors were concerned about the problem of overfitting (52).

## 4 Discussion

In this systematic literature survey we have discussed the use of machine learning and natural language processing methods for the detection of eating disorders. Our survey was conducted using the PRISMA framework (17). Our results have shown that many studies focus on the detection of anorexia, or eating disorders in general (see **Figure 7**). We have also seen that there was more work over the last couple of years, indicating a growing interest in the topic (as shown in **Figure 3**). Whereas most publications were from institutions in the USA and Spain, work from other countries including Mexico, France and Canada was also identified, as shown in **Figure 4**. Nevertheless, our work has shown that most research efforts have only been applied to the English language. Given the relevance of local languages for mental health diagnostics and treatment (15), it is thus necessary for future research to address other languages. With regard to the machine learning and feature extraction methods being applied, a comparison turned out to be challenging due to the diverse nature of the datasets and approaches used. The proposed approaches were classified into different categories, including classical machine learning, deep learning, a combination of classical and deep learning, the use of large language models, as well as other approaches. Several studies used f1-score as a common measure, reaching different performances ranging from 0.67 to 0.93. Overall, having a sufficient data quality and quantity was often seen as a major limitation of the approaches. Since 2017, the eRisk challenge has included two tasks pertaining to the early detection of Eating Disorders. In both 2018 and 2019, the task involved the early detection of signs of anorexia [see e.g., Losada et al. (26)]. In contrast, the 2022 iteration introduced a novel task centered on measuring the severity of eating disorders (27). This task diverged from the previous ones in that no labeled training data was supplied to participants, meaning that participants could not evaluate the quality of their models’ predictions until test time. The objective task was to assess a user’s level of eating disorder severity through analysis of their Reddit posting history.

Given the composition of both the eRisk lab and the SMHD dataset (50) predominantly with social media data, it is notable that an overwhelming majority (93%) of the studies in our analysis employ this data type. This underscores the widespread reliance on social media sources in modern research methodologies. This finding confirms the results of Zhang et al. (16) who found that among 399 papers applying NLP methods for the identification of mental health problems, 81% consisted of social media data.

It is worth mentioning that we came across two types of use cases in the studies. Many studies focus on the individual’s expression of their behavior and feelings with regard to eating disorders. Some studies, namely Choudhury (30) and Chancellor et al. (49), investigate the wording of pro-anorexia or pro-eating disorders communities on social media and online forums. Such communities promote disordered eating habits as acceptable alternative lifestyles (49). Whereas in many of the studies the technologies target support for clinical professionals, in these cases other applications such as content moderation are in the foreground.

In the realm of data collection for eating disorder research, manual labeling of datasets has been a common approach, with various strategies employed. For instance, Zhang et al. (45) relied on the voluntary efforts of 31 individuals to meticulously annotate 8554 data points encompassing 38 symptoms related to MD (Mental Disorders). Other studies took different routes, combining expert knowledge with input from non-expert annotators^9^ (38), or solely relying on domain experts (46). In some cases, researchers have employed machine learning algorithms to automatically annotate their datasets and subsequently validated the results with input from human labelers (44). The majority of datasets underwent annotation by non-expert human annotators, as seen in studies conducted by (79, 40, 34, 41).

Our review revealed few instances of Large Language Models (LLMs) application (10, 11, 19, 30, 38, 43, 44, 45, 49, 50, 61, 67, 73, 74, 79, 80). Despite this, the rising adoption of technologies like MentalBERT (77) and MentaLLama (81), alongside traditional machine and deep learning approaches, is notable. This trend, driven by the impressive efficacy of LLMs in natural language processing, is expected to continue on. As these technologies evolve and become more accessible, we anticipate their increased utilization in this field of research, enhancing computational model accuracy and efficiency.

Based on the identified limitations in the selected studies, we infer the following focus topics that we suggest for future work in the field of using natural language processing and machine learning in ED research:

- Data Quantity and Quality: how can more high-quality data be created and shared, while respecting the ethical and privacy limitations of such sensitive data?
- Involvement of Clinical Professionals: how can machine learning engineers and clinical professionals work together more closely?
- More Diversity in Data: How can the diversity of the population in the used datasets be increased to avoid bias in the classification?
- Local Languages: How can the proposed methods be extended to local languages other than English?

In conclusion, based on the studies investigated in this literature survey, there is potential for further development and in the long-term a novel tool support for clinical professionals based on text data.

## Author contributions

GM: Formal analysis, Writing – review & editing, Writing – original draft, Visualization, Investigation, Data curation. AP: Formal analysis, Writing – review & editing, Writing – original draft, Validation, Supervision, Methodology, Conceptualization. MK-B: Conceptualization, Funding acquisition, Methodology, Project administration, Supervision, Writing – original draft, Writing – review & editing.

## Footnotes

1
https://icd.who.int/browse10/2019/en#/F50

2 Valence is a measure of the emotional intensity or positivity/negativity associated with a word.

3 See e.g., https://digitalreality.ieee.org/publications/what-is-augmented-intelligence

4
https://erisk.irlab.org/

5
https://www.corc.uk.net/media/1273/ede-qquesionnaire.pdf

7
https://www.corc.uk.net/media/1273/ede-qquesionnaire.pdf

8 A content or an activity that promotes or encourages eating disorders (EDs).

9 individuals who lack specialized domain knowledge or expertise in the subject matter.
